# In search of the most cost‐effective monitoring strategy for vestibular schwannoma: A decision analytical modelling study

**DOI:** 10.1111/coa.13326

**Published:** 2019-04-11

**Authors:** Mirre Scholte, Mayke A. Hentschel, Gerjon Hannink, Henricus P. M. Kunst, Stefan C. Steens, Maroeska M. Rovers, Janneke P. C. Grutters

**Affiliations:** ^1^ Department of Operating Rooms, Radboud Institute of Health Sciences Radboud University Medical Center Nijmegen The Netherlands; ^2^ Department of Otolaryngology, Radboud Institute of Health Sciences Radboud University Medical Center Nijmegen The Netherlands; ^3^ Department of Otolaryngology Maastricht University Medical Center Maastricht The Netherlands; ^4^ Department of Radiology and Nuclear Medicine Radboud University Medical Center Nijmegen The Netherlands; ^5^ Department for Health Evidence, Radboud Institute of Health Sciences Radboud University Medical Center Nijmegen The Netherlands

**Keywords:** acoustic neuroma, cost‐effectiveness analysis, magnetic resonance imaging, monitoring, vestibular schwannoma, wait and scan

## Abstract

**Objectives:**

To assess the cost‐effectiveness of frequently used monitoring strategies for vestibular schwannoma (VS).

**Design:**

A state transition model was developed to compare six monitoring strategies for patients with VS: lifelong annual monitoring; annual monitoring for the first 10 years after diagnosis; scanning at 1‐5, 7, 9, 12, 15 years after diagnosis and subsequently every 5 years; a personalised monitoring strategy for small and large tumours; scanning at 1, 2 and 5 years after diagnosis and no monitoring. Input data were derived from literature and expert opinion. Quality‐adjusted life years (QALYs) and healthcare costs of each strategy were modelled over lifetime. Net monetary benefits (NMBs) were calculated to determine which strategy provided most value for money. Sensitivity analyses were performed to address uncertainty.

**Results:**

Omitting monitoring is least effective with 18.23 (95% CI 16.84‐19.37) QALYs per patient, and lifelong annual monitoring is most effective with 18.66 (95% CI 17.42‐19.65) QALYs. Corresponding costs were €6526 (95% CI 5923‐7058) and €9429 (95% CI 9197‐9643) per patient, respectively. Lifelong annual monitoring provided the best value with a NMB of €363 765 (339 040‐383 697), but the overall probability of being most cost‐effective compared to the other strategies was still only 23%. Sensitivity analysis shows that there is large uncertainty in the effectiveness of all strategies, with largely overlapping 95% confidence intervals for all strategies.

**Conclusions:**

Due to the largely overlapping 95% confidence intervals of all monitoring strategies for VS, it is unclear which monitoring strategy provides most value for money at this moment.

**Keypoints**

- The aim was to determine the added value of different monitoring strategies for VS.
- The majority of patients with VS are nowadays observed through a monitoring strategy. In clinical practice, multiple monitoring protocols are used alongside each other, often lacking evidence of effectiveness.
- Six monitoring strategies were compared: lifelong annual monitoring; annual monitoring for the first 10 years after diagnosis; scanning at 1‐5, 7, 9, 12, 15 years after diagnosis and subsequently every 5 years; a personalised monitoring strategy for small and large tumours; scanning at 1, 2 and 5 years after diagnosis and no monitoring at all.
- All included monitoring strategies had a low probability to be most cost‐effective compared to other included strategies. Ranging from 23% for lifelong annual monitoring to 11% for no monitoring.
- Due to the largely overlapping 95% confidence intervals of all monitoring strategies for VS, it is unclear which monitoring strategy provides most value for money at this moment.

## 1 INTRODUCTION

Vestibular schwannomas (VSs) are benign, slow‐growing tumours originating from Schwann cells of the vestibular part of the eighth cranial nerve.^1^ They represent 6% of all intracranial tumours.^2^ Patients with sporadic VS most commonly present between their 40s and 60s, some with small intracanalicular tumours and others with larger extrameatal tumours expanding into the cerebellopontine angle.^3^

The possibility to observe tumour development with magnetic resonance imaging (MRI) has led to the adoption of a “wait and scan” or “monitoring” policy in addition to treatment options; microsurgery and stereotactic radiosurgery (SRS).^3^, ^4^, ^5^ Currently, treatment is mainly indicated for large and/or growing tumours. Due to monitoring, it is known that approximately two thirds of VSs grow slowly or do not grow at all, which resulted in a decline of initial treatment and an increase in conservative management.^6^, ^7^ At present, it is not possible to predict which VSs pose a threat and which can be safely left without intervention; therefore, all patients undergo a monitoring strategy with extensive MRI scanning.

Magnetic resonance imaging scans are costly and, with a large proportion of patients in a monitoring strategy, contribute significantly to the high costs involved with VS.^8^ Multiple monitoring protocols are used alongside each other, often lacking evidence of effectiveness.^9^, ^10^ Therefore, a cost‐effectiveness model was developed to determine the added value of monitoring strategies for VS.

## 2 METHODS

### 2.1 Ethical considerations

This modelling study was based on published literature and did not involve human subjects, and therefore, ethical approval or informed consent was not required.

### 2.2 Model development

To simulate the follow‐up of patients in a monitoring strategy, we developed a state‐transition model in which we simulated costs and quality of life associated with multiple monitoring strategies for VS. The target population comprised VS patients who were initially assigned to the monitoring strategy, that is tumours smaller than Koos 4 at time of diagnosis or small Koos 4 tumours without symptoms of brainstem compression (hydrocephalus and symptoms caused by cranial nerve failure, eg, swallowing problems).^11^ The model starts at the age of 55, the mean age of diagnosis.^12^ We assumed every patient was eligible for MRI, and loss to follow‐up did not occur. Based on clinical guidelines and expert interviews, the model was designed in a way that it resembles the clinical situation.

**Figure 1:**
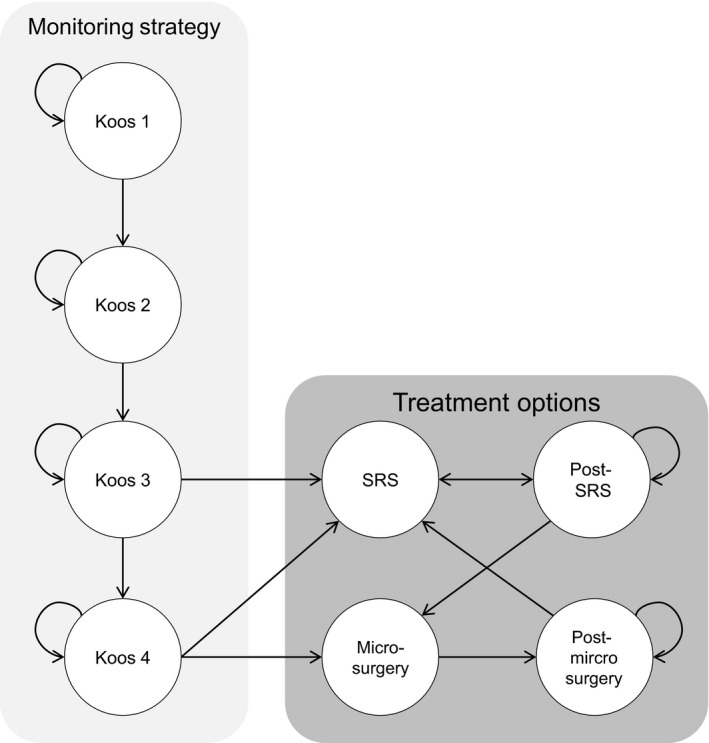
Influence diagram of the Markov model. Patients could enter the model via one of the Koos states in the monitoring strategy. Koos 1 corresponds to an intracanalicular VS, Koos 2 to an extracanalicular VS without brainstem contact, Koos 3 to VS with brainstem contact and Koos 4 corresponds to VS that compresses the brainstem. When tumour growth was present, patients entered the next Koos state. In case of Koos state 3 and 4, patients exited the monitoring strategy when tumour growth was detected on MRI. Leaving the monitoring strategy meant transition to one of the treatment options; stereotactic radiosurgery (SRS) or microsurgery. After treatment, patients were monitored for tumour growth. If tumour growth was detected after treatment, patients could receive additional treatment. The health state “dead” is not displayed, but could be entered from all health states

A state‐transition model describes the conditions that patients can be in (health states), how they can move among such states (transitions) and how likely such moves are (transition probabilities). Health states in the model were “Koos 1,” “Koos 2,” “Koos 3,” “Koos 4,” “microsurgery,” “post‐microsurgery,” “SRS,” “post‐SRS” and “dead” (Figure 1). Patients were assumed to enter the model via one of the Koos states. The Koos states represented different tumour sizes in the monitoring strategy, for patients who were not treated for their VS. We added treatment options to model the consequences of tumour growth. Tumour growth was defined as growth to the next Koos state, and in case of a Koos 4 tumour as growth to a Koos 4 tumour with brainstem compression. Growth within a Koos state and tumour shrinkage were not considered growth. Small‐ and medium‐sized tumours (Koos 1 and 2) which showed growth continued to be monitored without treatment. Patients received SRS when growth from Koos 2 to 3 was detected and SRS or microsurgery when growth to Koos 4 was detected. We assumed that when a growing VS was not detected and treated in time, the patient would visit the hospital with symptoms of brainstem compression and would then receive microsurgical treatment. Quality of life was lower in the year prior to surgical treatment.

The model had a cycle length of 1 year with a lifelong time horizon. We applied discount rates to costs and effects, to adjust future costs and effects to present values. A discount rate of 4% was applied to costs and 1.5% to quality‐adjusted life years (QALYs), according to Dutch guidelines.^13^

### 2.3 Model validation

We verified the model's validity using the AdViSHE checklist.^14^ This checklist covers five aspects of validation: conceptual model, input data, computerised model and operational validation and other validation techniques. The conceptual model was tested on its face validity (the model's appropriateness to represent the clinical process/disease) by consulting otolaryngology, radiology, SRS and neurosurgery experts in the Netherlands. The conceptual model was also cross‐validated with other VS models in literature; however, no specific health‐economic models for monitoring strategies were found. Face validity of the input data was tested by consulting the above‐mentioned experts. The computerised model was validated by extreme value testing, to detect possible coding errors. Operational validity was tested by discussing the model outcomes with the above‐mentioned experts. In addition, sensitivity analyses were performed to validate the outcomes with alternative input data. Last, the model was checked for inconsistencies by an independent expert.

### 2.4 Strategies

We modelled multiple monitoring strategies for the follow‐up of VS: lifelong annual monitoring; annual monitoring for the first 10 years after diagnosis; scanning at 1‐5, 7, 9, 12, 15 years after diagnosis and subsequently every 5 years; a personalised monitoring strategy for small and large tumours; scanning at 1, 2 and 5 years after diagnosis and no monitoring. In the personalised monitoring strategy, small tumours (Koos 1 and 2) are monitored 1‐3, and 5 years following diagnosis and large tumours (Koos 3 and 4) are monitored 1‐5, 8, 11 and 16 years following diagnosis. A strategy without monitoring was modelled to evaluate the consequences of omitting monitoring, since there is discussion about the added value of current monitoring strategies.^9^ In the no monitoring strategy, we assumed that if symptoms of brainstem compression occurred, patients would visit the hospital and undergo microsurgery. We used conservative assumptions for this strategy: patients acquired brainstem compression when ≥2 mm growth in Koos 4 occurred, quality of life was low for 1 year when brainstem compression occurred and costs, and consequences of microsurgery were adapted to large tumours by assuming a complication rate of 25% instead of 12.5%.^15^

### 2.5 Transition probabilities

Probabilities were derived from literature and expert opinion (Table 1). All expert‐based values were confirmed by at least two experts. Key inputs were the initial probabilities that divided patients over the Koos states, which were derived from Stangerup et al,^3^ that is 34.7%, 32.2%, 32.2% and 0.9% for Koos 1, Koos 2, Koos 3 and Koos 4, respectively. Transition among Koos states was defined by the probability of tumour growth to the next Koos state and the probability to have ≥2 mm growth in Koos 4. These probabilities were derived from a large (n = 1217) retrospective study conducted in our hospital (Figure S1). For each Koos state, follow‐up was at least 9 years. Thereafter, we assumed tumour growth not to occur.

**Table 1:** Model parameters

Parameter	Value^a^	Source
Probabilities		
Koos 1	0.347 (*α* 112, *β* 211)	Stangerup et al^3^
Koos 2	0.322 (*α* 104, *β* 219)	Stangerup et al^3^
Koos 3	0.322 (*α* 104, *β* 219)	Stangerup et al^3^
Koos 4	0.009 (*α* 3, *β* 320)	Stangerup et al^3^
Dead	Standard mortality rates	Statistics Netherlands^24^
Tumour growth to the next Koos state	Figure S1	Patient cohort Radboudumc
SRS after growth in the Koos 3 state	1.00	Expert opinion
Microsurgery after growth in the Koos 4 state	0.900	Expert opinion
Microsurgery complications	0.125	Sughrue et al^15^
Death as a consequence of microsurgery	0.002	Sughrue et al^15^
Death as a consequence of SRS	0	Klijn et al^25^
Growth after microsurgery	0.003	Godefroy et al^26^
Growth after SRS	0.006	Klijn et al^25^
Microsurgery in case of growth in the post‐SRS state	0.400	Expert opinion
SRS in case of growth in the post‐microsurgery state	1.00	Expert opinion
Costs		
Consultation—tertiary hospital	€167	Dutch Guideline for costing research^13^
Consultation—general hospital	€82	Dutch Guideline for costing research^13^
MRI brain	€211	Dutch Guideline for costing research^13^
Microsurgery—uncomplicated	€10 406	Dutch health care administration
Microsurgery—complicated	€13 068	Dutch health care administration
SRS	€8876	Dutch health care administration
Post‐microsurgery	€151 (90% of all patients are followed in a tertiary hospital after microsurgery)	Expert opinion
Post‐SRS	€153 (85% of all patients are followed in a general hospital	Expert opinion
Utilities		
Monitoring strategy	Year 1‐3: 0.831 (SD 0.244) Year 4‐6: 0.826 (SD 0.244) Year 7‐9: 0.821 (SD 0.244) Year 10‐12: 0.816 (SD 0.244) Year 13 and onwards: 0.811 (SD 0.244)	Gait et al^19^, Godefroy et al^18^
Symptoms of brainstem compression	0.537 (SD 0.283)	Turel et al^27^
First year after microsurgery	0.688	Gait et al^19^, Sughrue et al^15^
First year after SRS	0.789	Gait et al^19^, Klijn et al^25^
Post‐microsurgery	0.789	Godefroy et al^28^
Post‐SRS	0.811	Varughese et al^29^
Dead	0	

MRI, magnetic resonance imaging; SD, standard deviation; SRS, stereotactic radiosurgery.

a β‐distributions were assigned to some of the parameters for use in the probabilistic sensitivity analysis. The characteristics of the β‐distribution are presented between brackets, either as an SD or as an *α* and *β* value (where *α* represents the number of events in a sample and *β* the number of non‐events).

### 2.6 Costs

The cost analysis was performed from a healthcare perspective, meaning all healthcare costs were included. Costs were assessed in Euros (€) and based on the 2017 price level. When available, costs were derived from the Dutch guideline for costing research.^13^ Otherwise, unit costs were obtained from hospital fees. Key costs were consultation costs, €167 for tertiary hospitals and €82 for general hospitals, and MRI scans of €211. Complication costs are included in the total costs of microsurgery and SRS. To determine annual costs after microsurgery or SRS, a scanning protocol with scans at 1‐5, 7, 9, 12, 15 years after microsurgery or SRS and subsequently every 5 years was assumed as this is the current protocol in our hospital (Table 1).

### 2.7 Effects

Effectiveness was measured in QALYs, which is a combination of quality of life (utility) and survival. A utility reflects quality of life on a 0‐1 scale, with 0 representing death and 1 representing full health. Most quality of life values for VS patients in literature are derived from the SF‐36 questionnaire. We used an algorithm to construct a utility value from the domain scores of the SF‐36 questionnaire (Table 1).^16^, ^17^ Quality of life in the monitoring strategy was assumed to gradually decline, since symptoms of asymmetric sensorineural hearing loss, tinnitus and vertigo often progress over time.^18^ In the year of treatment, a lower utility was assumed due to potential complications (by calculating the weighted mean of treatment associated complications and corresponding utilities).

### 2.8 Analysis

A hypothetical cohort of 1000 patients was sent through the model to determine mean expected costs and effects (QALYs) per patient for each strategy. We compared the monitoring strategies to each other by calculating the average costs per QALY. We also calculated the net monetary benefit (NMB), which represents the value of a strategy in monetary terms. The strategy with the highest NMB represents the most cost‐effective strategy. The NMB is calculated by multiplying the gained QALYs by the threshold value minus costs of the monitoring strategy. We used a threshold value of €20 000 per QALY, as recommended by the Dutch guidelines.^13^

We performed a scenario analysis in which an alternative treatment scheme is used. In this scheme, growing Koos 2 tumours which were initially diagnosed as Koos 1 were treated with SRS when detected by MRI. We also performed a probabilistic sensitivity analysis with 10 000 simulations to investigate sampling uncertainty concerning the parameters in the model. We did this for important variables: initial probabilities, growth rates, utilities in the monitoring strategy and the utility of brainstem compression (Table 1). The percentile method was used to calculate 95% confidence intervals (CIs) from simulations. Simulation results are presented in cost‐effectiveness planes and cost‐effectiveness acceptability curves (CEACs). All analyses were conducted using TreeAge Pro 2015 (TreeAge Software, Inc), and percentiles were calculated in Excel 2007 (Microsoft).

## 3 RESULTS

We assessed the cost‐effectiveness of multiple monitoring strategies for the follow‐up of VS. Omitting monitoring is least effective with on average 18.23 (95% CI 16.84‐19.37) QALYs while lifelong annual monitoring is most effective with 18.66 (95% CI 17.42‐19.65) QALYs per patient. Overlapping 95% CIs were found regarding the effectiveness of all six monitoring strategies (Table 2). Lifelong annual monitoring was the most expensive strategy with average costs of €9429 (95% CI 9197‐9643) per patient. Omitting monitoring is the least expensive strategy with average costs of €6526 (95% CI 5923‐7058) per patient, which are mainly treatment costs.

**Table 2:** Outcomes

Strategy	Costs (€)	Effects (QALYs)	NMB (€)
1. Lifelong annual monitoring	9429 (9197‐9643)	18.66 (17.42‐19.65)	363 765 (339 040‐383 697)
2. Annual monitoring for the first 10 y after diagnosis	8684 (8297‐9033)	18.54 (17.26‐19.55)	362 174 (336 438‐382 311)
3. Scans at 1‐5, 7, 9, 12, 15 after diagnosis and subsequently every 5 y	8585 (8232‐8911)	18.52 (17.27‐19.54)	361 788 (336 809‐382 335)
4. Personalised monitoring strategy for small and large tumours	8149 (7708‐8552)	18.46 (17.15‐19.49)	360 986 (335 032‐381 638)
5. Scans at 1, 2 and 5 y after diagnosis	8032 (7588‐8439)	18.44 (17.12‐19.47)	360 774 (334 483‐381 507)
6. No monitoring	6526 (5923‐7058)	18.23 (16.84‐19.37)	358 168 (330 371‐380 908)

NMB, net monetary benefit; QALY, quality‐adjusted life year.

Lifelong annual monitoring had the highest NMB, €363 765 (95% CI 339 040‐383 697), and therefore provides most value for money (ie, the strategy gained most QALYs at a price that we are willing to pay as society). This strategy was followed by annual monitoring for the first 10 years with an NMB of €362 174 (95% CI 336 438‐382 311). The strategy with the lowest NMB, representing the least cost‐effective strategy, was no monitoring with an NMB of €358 168 (95% CI 330 371‐380 908). Although this strategy was least expensive, it also gains the least QALYs. The savings in this strategy do not weigh up against the QALYs lost, hence the lower NMB of this strategy. The 95% CIs for the NMBs were largely overlapping for all strategies (Table 2).

Using an alternative treatment scheme (in which growing Koos 2 tumours are treated with SRS when detected) resulted in additional costs, as more patients received treatment. Treatment outcomes for Koos 2 and Koos 3 tumours were the same, and therefore, no differences in quality of life were expected in case of annual monitoring. However, in other monitoring strategies, treating growing Koos 2 tumours resulted in higher quality of life as brainstem compression is prevented (Table 3). Therefore, alternative treatment is cost‐effective compared to treating only Koos 3 and 4 in these monitoring strategies.

**Table 3:** Additional costs and effects of using an alternative treatment scheme

Strategy	Additional costs^a^ (€)	Additional effects^a^ (QALYs)	Incremental NMB^b^ (€)
1. Lifelong annual monitoring	199	0.00	−199
2. Annual monitoring for the first 10 y after diagnosis	174	0.01	26
3. Scans at 1‐5, 7, 9, 12, 15 after diagnosis and subsequently every 5 y	114	0.03	486
4. Personalised monitoring strategy for small and large tumours	82	0.03	518
5. Scans at 1, 2 and 5 y after diagnosis	88	0.03	512
6. No monitoring	0	0.00	0

NMB, net monetary benefit; QALY, quality‐adjusted life year.

In this strategy, growing Koos 2 tumours are treated with SRS when detected. We calculated the additional costs and effects for each monitoring strategy, compared to the same monitoring strategy in the base case analysis.

a Outcomes of this sensitivity analysis were compared to the base case analysis, for each monitoring strategy.

b A positive incremental NMB indicates that the strategy is cost‐effective compared to the base case analysis.

**Figure 2:**
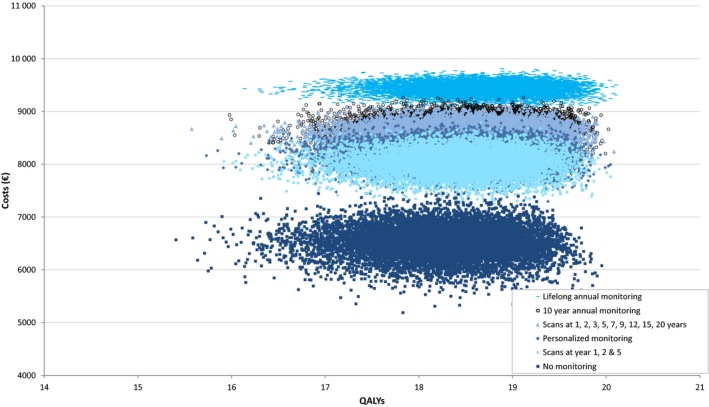
Outcomes of the probabilistic sensitivity analysis. This analysis quantifies the level of confidence of the model's conclusions. All six monitoring strategies are displayed. Every dot represents the outcome of one analysis

**Figure 3:**
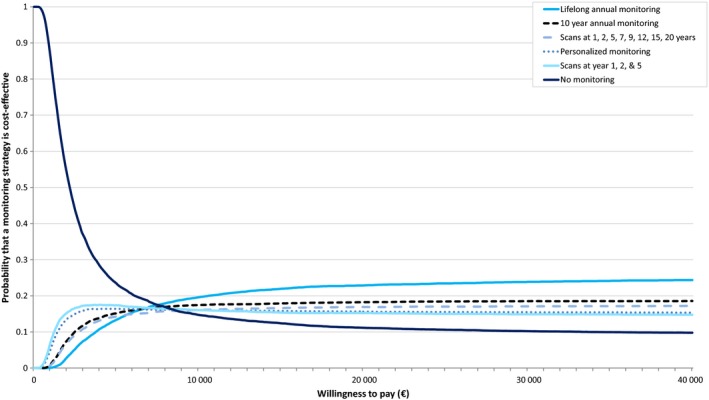
Cost‐effectiveness acceptability curve. This graph shows the probability that one of the strategies is most cost‐effective for different willingness to pay values. The willingness to pay represents an estimate of what we might be prepared to pay for the health benefit

In Figure 2, the incremental results of the probabilistic sensitivity analysis are shown. There is uncertainty in the effectiveness of all strategies, resulting in largely overlapping 95% CIs for QALYs and NMBs. The CEAC shows that all strategies have a relatively low probability to be most cost‐effective due to large uncertainty in the results (Figure 3). At a threshold of €20 000 per QALY, lifelong annual monitoring has a 23% probability to be the most cost‐effective strategy, which is higher than annual monitoring for the first 10 years (18%), scans at 1‐5, 7, 9, 12, 15 years after diagnosis and subsequently every 5 years (16%), personalised monitoring (16%), scans at 1, 2 and 5 years after diagnosis (15%) and no monitoring (11%).

## 4 DISCUSSION

### 4.1 Synopsis of key findings

We assessed the cost‐effectiveness of multiple monitoring strategies for VS. Omitting monitoring is least effective with on average 18.23 (95% CI 16.84‐19.37) QALYs while lifelong annual monitoring is most effective with 18.66 (95% CI 17.42‐19.65) QALYs per patient. Corresponding costs were €6526 (95% CI 5923‐7058) and €9429 (95% CI 9197‐9643) per patient, respectively. Lifelong annual monitoring appeared to be most cost‐effective with a NMB of €363 765 (95% CI 339 040‐383 697). An alternative treatment scheme in which growing Koos 2 tumours are also treated was cost‐effective when patients were not annually monitored. Sensitivity analysis shows that there is large uncertainty regarding the effectiveness of all strategies.

### 4.2 Strengths and limitations

To our knowledge, this is the first study to investigate cost‐effectiveness of multiple monitoring strategies of VS. Others have studied cost‐effectiveness of treatment strategies such as SRS or microsurgery.^19^, ^20^, ^21^ However, the majority of patients with VS are nowadays observed through a monitoring strategy. In clinical practice, multiple monitoring strategies are used alongside each other, often lacking evidence of (cost‐)effectiveness.^9^ We therefore studied the cost‐effectiveness of different monitoring strategies.

Some potential limitations should also be discussed. First, costs are based on Dutch healthcare prices and may therefore slightly differ from other countries. The same applies to expert opinions, which can differ between hospitals and countries. We expect differences in exact costs and effects for other countries, but a similar trend. Given the detailed presentation of the model and its input parameters, those interested can assess the transferability of the results to their specific situation.

Second, we included VSs of all sizes into the monitoring strategy as this represents current practice in the Netherlands. Only 5% of tumours receive treatment directly following diagnosis. Monitoring for large tumours is more controversial, since the risk of brainstem compression is larger. When a less conservative management strategy is used, relatively smaller tumours will be included in the monitoring strategy with less severe consequences of undetected tumour growth. In this case, less intensive monitoring strategies would become more cost‐effective.

Third, the construction of QALYs in this model required generic quality of life scores. We used the EQ‐5D or SF‐36 questionnaires, which are relatively insensitive for hearing problems compared to disease‐specific questionnaires such as the PANQOL. However, there is currently no algorithm available that converts PANQOL outcomes to generic utility scores. Another generic questionnaire, the Health Utilities Index (HUI), does allow for the calculation of utility scores. Because it is more sensitive for hearing problems, the HUI seems more suitable to measure generic quality of life in patients with VS.^22^ Unfortunately, we were unable to find utility scores measured by HUI for use in our model.

Last, transition from one health state to another in the monitoring strategy was based on the probability for a tumour to grow to the next Koos state. We chose these Koos states since they report clearly defined cut‐off points, take tumour size and localisation in relation to other structures into account, and have clearly defined consequences (ie, recommended treatment).^23^ We acknowledge that by using the Koos states as cut‐off points, we were not able to detect growth within a Koos state. However, treatment options only change in case of progression to a next Koos state; therefore, missing growth within a Koos state does not have consequences for treatment.

### 4.3 Implications for clinical practice

Currently, large differences in the management of VS are present. Multiple monitoring strategies are used alongside each other, without clear evidence of effectiveness.^9^ In this analysis, we assessed the cost‐effectiveness of several monitoring strategies. Looking at point estimates, lifelong annual monitoring seems most cost‐effective. VSs are treated in time in this strategy, preventing serious consequences of brainstem compression.

However, the 95% CIs are largely overlapping with all other strategies. Based on the currently available evidence, the probability that lifelong annual monitoring is cost‐effective is only 23%. This implies that if lifelong annual monitoring is implemented, the probability that this is the wrong decision is 77%. As there is considerable uncertainty surrounding this decision, it might be better to wait for more evidence before we spend money on extensive monitoring strategies.

As shown in Figures 2 and 3, cost‐effectiveness outcomes are very uncertain with probabilities for a strategy to be most cost‐effective ranging from 11% to 23%. The uncertainty is mainly caused by uncertain effectiveness outcomes, due to the use of suboptimal effectiveness measures in literature and small sample sizes of study populations. Larger, high‐quality studies that investigate quality of life in VS patients assigned to a monitoring strategy using the HUI questionnaire are needed to achieve reliable effectiveness estimates. When research is initiated on this topic, a no monitoring strategy should be included. We used conservative assumptions for the no monitoring strategy; therefore, we might be underestimating the cost‐effectiveness of this strategy in this paper. Also, many patients remain in a monitoring strategy for life without needing treatment; therefore, a no monitoring strategy could considerably lower the costs of monitoring.

In conclusion, due to the largely overlapping 95% CIs of all monitoring strategies for VS, it is unclear which monitoring strategy provides most value for money at this moment.

## CONFLICT OF INTEREST

There are no conflicts of interest.

## Data Availability

The data that support the findings of this study are available from the corresponding author upon reasonable request.
