# Supplementary material for: In search of the most cost‐effective monitoring strategy for vestibular schwannoma: A decision analytical modelling study
Source: Clin Otolaryngol. 2019 Apr 11;44(4):525–33. doi: 10.1111/coa.13326 (PMC6850121; doi:10.1111/coa.13326)
Supplement: Supplementary file 1 [file COA-44-525-s001.pdf]

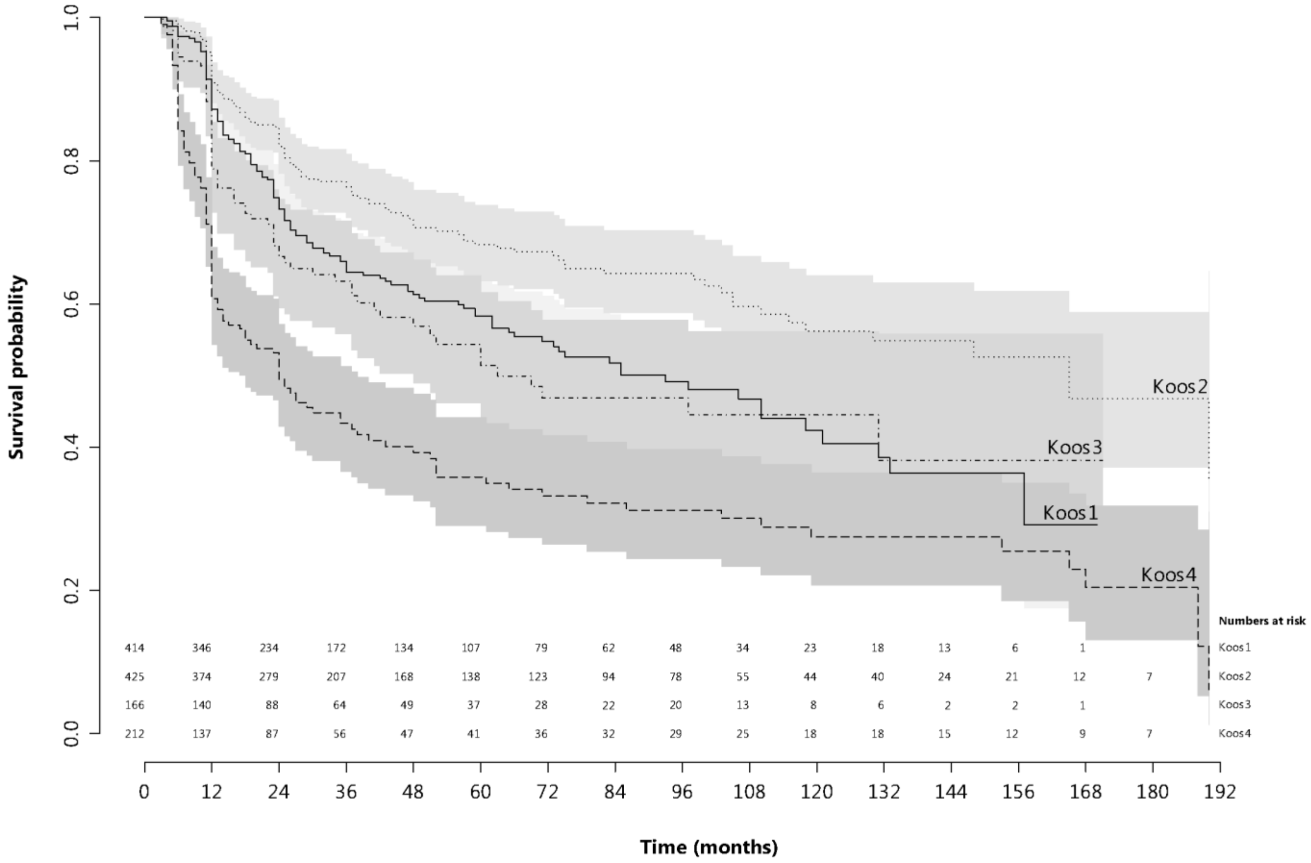

Kaplan-Meier estimates of VS growth rates to the next Koos state, displayed per Koos state at diagnosis. We used a retrospective cohort (n=1217) of patients diagnosed with VS in the Radboudumc between 1990 and 2016. Patients with a unilateral VS, assigned to the monitoring strategy that had at least one follow-up MRI available were included. Transition among Koos states was defined by the probability of tumour growth to the next Koos state and the probability to have ≥2mm growth in Koos 4. Missing data were assumed to be missing at random. Missing data were imputed by multiple imputation using chained equations to create 25 imputed data sets. One randomly selected set was included in our model. We included the probability of tumour growth when sample sizes were ≥10 patients, thereafter growth was assumed not to occur. (KM: Kaplan-Meier).
